# Role of Mitogen-Activated Protein Kinase Pathways in Multifactorial Adverse Cardiac Remodeling Associated with Metabolic Syndrome

**DOI:** 10.1155/2013/367245

**Published:** 2013-01-09

**Authors:** Mohamed Asrih, François Mach, Alessio Nencioni, Franco Dallegri, Alessandra Quercioli, Fabrizio Montecucco

**Affiliations:** ^1^Division of Cardiology, Geneva University Hospital, Faculty of Medicine, Foundation for Medical Researches, 64 Avenue de la Roseraie, 1211 Geneva, Switzerland; ^2^Department of Internal Medicine, University of Genoa, V.le Benedetto XV 6, 16132 Genoa, Italy; ^3^First Clinic of Internal Medicine, Department of Internal Medicine, University of Genoa, V.le Benedetto XV 6, 16132 Genoa, Italy

## Abstract

Metabolic syndrome has been widely associated with an increased risk for acute cardiovascular events. Emerging evidence supports metabolic syndrome as a condition favoring an adverse cardiac remodeling, which might evolve towards heart dysfunction and failure. This pathological remodeling has been described to result from the cardiac adaptive response to clinical mechanical conditions (such as hypertension, dyslipidemia, and hyperglycemia), soluble inflammatory molecules (such as cytokines and chemokines), as well as hormones (such as insulin), characterizing the pathophysiology of metabolic syndrome. Moreover, these cardiac processes (resulting in cardiac hypertrophy and fibrosis) are also associated with the modulation of intracellular signalling pathways within cardiomyocytes. Amongst the different intracellular kinases, mitogen-activated protein kinases (MAPKs) were shown to be involved in heart damage in metabolic syndrome. However, their role remains controversial. In this paper, we will discuss and update evidence on MAPK-mediated mechanisms underlying cardiac adverse remodeling associated with metabolic syndrome.

## 1. Introduction

The prevalence of metabolic syndrome is rapidly increasing in the western world [1]. Metabolic syndrome has been defined as a cluster of multiple disorders including insulin resistance, abdominal obesity, dyslipidemia, increased blood pressure, hypercholesterolemia, and proinflammatory state [2]. Several definitions have been historically proposed during the last decades, also including oxidative stress, leptin resistances and endothelial dysfunction as key pathophysiological mechanisms contributing to the increase of cardiovascular risk that affect metabolic syndrome patients [2, 3]. Considering these paradigms, it is clear that the metabolic syndrome is a fully heterogeneous construction, raising important scientific limitations for meta-analyses of clinical investigations. Although some common components (such as dyslipidemia, hypertension, and hyperglycemia) are recurrent in the different definitions [4], these other disorders might represent an important variable in the analysis of different cohorts. Several conditions included in the metabolic syndrome have been shown as strongly associated with an acceleration of atherogenesis and an increased incidence of acute ischemic events [1]. In addition, some processes (mainly systemic insulin resistance and inflammation) have been proposed to contribute to physiological organ remodelling and pathological damage in metabolic syndrome. In particular, different chronic adverse heart remodeling and the development of liver steatosis have been widely described [5, 6]. In this paper, we will focus on the pathophysiology of heart remodelling and damage during metabolic syndrome. Metabolism syndrome patients affected by diabetes are associated with altered myocardial substrate metabolism, which has emerged as an important contributor to the development of cardiomyopathy [7]. In diabetes and concomitant metabolic syndrome, an increased cardiac fatty acid metabolism and reduced glucose metabolism have been reported [7]. Although initially profitable, the rate of fatty acid uptake reaches a point where it exceeds the rate of fatty acid oxidation, thereby promoting the accumulation of lipids, resulting in lipotoxicity and associated cardiac dysfunction [8]. This leads to some complications, such as cardiac hypertrophy, which is identified as a cardiac pathological remodeling. However, cardiac remodeling does not necessarily refer to pathological adaptation of the myocardium. Indeed, short-term compensatory mechanisms are beneficial for the heart because it adapts cardiac output to physiological or pathological loading conditions such as exercise, hypertension, or aortic stenosis. In contrast, sustained overload leads to maladaptive and detrimental remodeling, as reported in detail by Buckberg and coworkers [9]. Altogether, these studies suggest that cardiac remodeling in metabolic syndrome depends on the different component disorders and might not be a disease *per se* but rather an adaptive response. 

 Several intracellular signaling pathways, continuously sensing the extracellular stimuli and modulating the different intracellular responses, have been investigated to characterize their role in cardiomyocyte modifications and potential injury associated with metabolic syndrome. Mitogen-activated protein kinases (MAPKs) are cytosolic signaling proteins that become activated after specific phosphorylation [10]. In response to wide extracellular stimuli, MAPKs have been shown to modulate various cellular processes, such as cell growth and cell size regulation [11]. Although not specifically performed in models of metabolic syndrome, *in vitro* studies using isolated cardiomyocytes have shown that MAPKs might be involved in cardiac hypertrophy via three traditional phases: (i) the activation of specific transmembrane proteins; (ii) intracellular signal transduction; (iii) the activation of cytosolic and nuclear events [12]. Since cardiac hypertrophy is characterized by increased cell size, it has been suggested that MAPKs might play a critical role in cardiac remodeling in hypertensive patients with metabolic syndrome. This appears to be achieved by modulating the activity of numerous transcription factors that target specific genes involved in structural response of the myocardium. In this paper, we will provide an overview on the role of MAPKs in the adverse cardiac remodeling that is associated with metabolic syndrome.

## 2. Different Structural Adverse Cardiac Remodeling in Metabolic Syndrome

Diagnostic criteria of metabolic syndrome (as indicated by ATP III classification [24]) have been reported to independently predict the development of diastolic dysfunction and cardiac hypertrophy [13, 19, 20] (Table 1). Cardiac hypertrophy is commonly defined as an increase in heart size or more particularly as an increase in ventricular size with or without increased wall thickness relative to body size [25]. This cardiac modification was shown to be a common alteration in subjects with different stages of obesity, which is one of the central features in metabolic syndrome [15, 16, 26–28]. These studies have used noninvasive methods such as echocardiography and magnetic resonance imaging (MRI) to assess cardiac adaptations in obese patients. The results indicate that obesity is associated with a high prevalence of cardiac hypertrophy, characterized by an enhancement of left ventricular cavity size as well as wall thickness. Moreover, it has been observed that wall thickness was increased to greater extent than left ventricular cavity size, revealing a concentric instead of eccentric cardiac hypertrophy. Indeed, computed tomography and MRI demonstrated that fat tissue deposits are well detectable within the heart of obese subjects and commonly accumulate anterior to the right ventricle [29–31]. Although noninvasive methods are useful to characterize cardiac size, structure, and function, they have limitations; in that they do not allow analyzing the biochemical composition of the hypertrophic heart in metabolic syndrome. Interestingly, postmortem studies confirmed the presence of cardiac hypertrophy and cardiac fat tissue deposits in obese patients [17, 18]. The amount of epicardial fat has been reported to be correlated with both visceral fat and the severity of ventricular hypertrophy [14]. Several studies have been conducted in animal models to better understand the cardiac adaptation in diabetes [32, 33]. Diabetes was associated with an increase in left ventricular internal dimension during diastole (LVIDD) and systole (LVIDS) in rats [34]. This adverse cardiac remodeling was independent of hypertension [34]. These results are consistent with a previous study, demonstrating that diabetic rats develop an eccentric left ventricular hypertrophy associated with a decreased cardiac systolic function, and related to impaired collagen turnover [35].

The Prospective Cardiovascular Münster (PROCAM) study, recruiting a cohort of 2754 males aged 40–65 years over a four-year period, showed that patients, who had either hypertension or diabetes, had a 2.5-fold increased risk of cardiovascular morbidity. However, when developing both, patients had an eightfold increase in cardiovascular risk. This multiplicative relevance was confirmed by a twentyfold increase of cardiovascular risk in patients with concomitant diabetes mellitus, hypertension, and abnormal lipid profile [36]. Since the metabolic syndrome might include all these disorders by definition, it is easy to understand that the alterations of an atherosclerotic targeted organ, such as the myocardium, might result from both acute and chronic ischemia. However, also in the absence of traditional atherosclerotic complications, the measurement of diastolic function worsened progressively during metabolic syndrome [13], indicating an adverse cardiac remodeling independent of cardiac necrosis and potentially related to the soluble mediators increased during the disease. In line with these results, different studies have confirmed the development of left ventricular diastolic dysfunction in subjects presenting metabolic syndrome [20, 21, 37, 38].

Interestingly, hypertensive patients with concomitant metabolic syndrome have been shown to present increased left ventricular mass and wall thickness as compared to patients exclusively affected by hypertension [39]. Furthermore, the authors showed that metabolic syndrome might induce an adverse cardiac remodeling via different pathophysiological mechanisms with a multiplicative effect. Metabolic syndrome patients had not only abnormal diastolic left ventricular relaxation, but also increased cardiac hypertrophy [40]. Cardiac hypertrophy predisposes individuals to cardiac arrhythmias, congestive heart failure, and diastolic dysfunction [41]. Consistently, several evidences have revealed a positive association between the metabolic syndrome and the severity of left ventricular hypertrophy [39, 40, 42]. Indeed, metabolic syndrome induces abnormal loading, which may favor the left ventricular hypertrophy. Since pathological cardiac hypertrophy has been associated with sudden cardiac death, heart failure, and stroke [43], it was proposed that this cardiac alteration might further increase cardiovascular risk in metabolic syndrome [44].

Increased myocardial fibrosis and stiffness have been also observed in animal models of obesity and metabolic syndrome [45]. Since collagen and fibrosis determine tissue compliance, cardiac deposition of this protein might promote left ventricular diastolic dysfunction and negatively affect diastolic function [46, 47]. Several other molecular mechanisms (i.e., insulin resistance aggravating asymptomatic myocardial inflammation and association between visceral obesity and myocardial adiposity) have also been suggested to potentially induce such cardiac structural alterations [48, 49]. 

Most importantly, the renin-angiotensin-aldosterone (RAA) system might also be involved cardiac fibroblast proliferation and collagen synthesis, thereby increasing cardiac fibrosis [50]. The active role for RAA system was confirmed by the finding that pharmacologic inhibition of this pathway ameliorates heart failure [51, 52], also in metabolic syndrome patients [53]. Thus, these studies suggest that RAA system could be a critical player underlying cardiac modifications in hypertensive metabolic syndrome patients.

## 3. Pathophysiological Mediators of Adverse Cardiac Remodeling in Metabolic Syndrome

Cardiac structural remodeling in metabolic syndrome might be due to different pathological triggers. Hypertrophic growth accompanies heterogeneous metabolic syndrome disorders, including not only diabetes and hypertension, but also coronary heart disease and ischemic cardiac remodeling. Mechanical alterations have been classically described as major causes inducing an adverse cardiac remodelling [54]. However, metabolic syndrome has been associated with left ventricular hypertrophy [40] independently of ischemic cardiac remodeling. Given the upregulation of several hormones and cytokines in metabolic syndrome patients [55], a potential role on cardiac remodeling has been proposed for these molecules [56]. In particular, a hormonal and inflammatory dysregulation might contribute to the development of cardiac hypertrophy and fibrosis [55]. For instance, increased aldosterone plasma levels in patients with metabolic syndrome [57, 58] might be directly associated with the development of left ventricular hypertrophy [59] or cardiac fibrosis [60]. Although clinical studies have reported that aldosterone induces left ventricular hypertrophy [61], the mechanism by which aldosterone promotes cardiac hypertrophy remains unclear. Okoshi and coworkers showed that aldosterone directly induced cardiac hypertrophy and atrial natriuretic peptide (ANP) mRNA expression (a molecular marker of cardiac hypertrophy) in neonatal rat ventricular myocytes [62]. These results were accompanied by enhanced activation of ERK1/2- and JNK-mediated pathways. This critical role of the ERK pathway in the development of cardiac hypertrophy (in response to endothelin-1) was also confirmed by the *in vitro* abrogation of cardiomyocyte hypertrophy in the presence of the pharmacological inhibitor of ERKs [63, 64].

On the other hand, aldosterone has been shown as a potent inducer of cardiac fibrosis [60, 65, 66]. Therefore, emerging evidence indicates a crucial role for aldosterone in maladaptive cardiac remodeling in metabolic syndrome [67, 68].

Considering the hypothesized inflammatory etiology of the metabolic syndrome [69], it was proposed that elevated circulating levels of cytokines, adipocytokines, and chemokines might actively regulate cardiac remodeling and via the activation of inflammatory signaling pathways [70–72]. Amongst several mediators, tumor necrosis factor (TNF) and interleukin-6 (IL-6) were shown to promote both insulin resistance [73] and cardiac hypertrophy [74], suggesting that inflammation in metabolic syndrome might be a central feature in atherogenesis as well as in cardiac hypertrophy. In particular, TNF expression, which was shown to be increased in response to pressure overload in the adult heart [75], might be one of the most important mediators of cardiac hypertrophy in metabolic syndrome with hypertension [76]. The molecular pathways potentially involved in metabolic syndrome-induced cardiac hypertrophy have been only partially investigated. To summarize, mechanical stress remains the main responsible of adverse cardiac remodelling in metabolic syndrome, such as hypertension. However, considering the potential dysregulation of inflammatory and hormonal systems, the cardiac pathophysiology in metabolic syndrome might be partially influenced also by these soluble molecules. In the following sections, we will review the pathophysiological role of MAPKs in cardiac remodeling in a general context and also particularly in metabolic syndrome.

## 4. Role of MAPK in Cardiac Remodeling

### 4.1. Extracellular Signal Regulated Kinases (ERKs)

One of the most studied MAPK pathways is the Ras/Raf/ERKs pathway. Extracellular stimuli such as stress or hormones activate diverse receptors at the cell surface, driving intracellular recruitment and activation of the guanosine triphosphate (GTP) small G-protein (Ras). This, in turn, induces Raf-1 kinase translocation to the plasma membrane and Raf-1-mediated phosphorylation of MEK proteins (MEK1 and 2). Thereafter, ERKs are activated by MEKs and regulate a large number of nuclear and cytosolic proteins [77] that directly modulate numerous intracellular processes.

For instance, pressure overload was shown to influence ERK-mediated intracellular signaling as well as extracellular matrix deposition within the heart [78]. In response to chronic pressure overload, cardiomyocytes start to grow leading to heart enlargement and hypertrophy. Pressure overload induced by transverse aortic constriction (TAC) in rodents was shown to mediate hypertrophic effect through ERK activation [79]. In addition, Esposito and coworkers showed that TAC procedure is associated with the activation of all three major MAPKs (ERK1/2, p38 MAPK, and JNK) in mice [80]. Consistent with the animal studies, clinical researches reported increased cardiac activation of ERK1/2, JNK, and p38 MAPK in failing human hypertrophic hearts [81]. In line with these results, ERK1/2 activation has been shown as a key element directly promoting cardiac hypertrophy [26, 82].

It was proposed that ERK1/2 induces the activation of various transcription factors by modulating their phosphorylation level, hence leading to hypertrophy. Indeed, using a model of phenylephrine-induced cardiac myocyte hypertrophy, Babu and coworkers revealed that ERK1/2 pathway is involved in Elk-1 upregulation in a model of phenylephrine-mediated hypertrophy [83]. Therefore, these studies suggest a central role for ERK in the pathophysiological development of cardiac hypertrophy. The molecular mechanisms downstream of this pathway remains to be clearly defined.

### 4.2. Janus Kinases (JNKs)

The hypertrophic effects of JNKs are still controversial. Wang and coworkers showed that specific activation of the JNK pathway was associated with hypertrophy in neonatal cardiomyocytes overexpressing MEK7 [84]. In line with these results, transfection of ventricular myocytes with MEK1 (a MAPK-activating JNK) leads to cardiac hypertrophy via JNK activation [85]. By contrast, transgenic mice selectively overexpressing MEK7 in the cardiac tissue were not shown to develop cardiac hypertrophy despite JNK1 and JNK2 upregulation [86]. Although these mice died from congestive heart failure, they had normal cardiomyocytes size and they did not develop ventricular hypertrophy. Importantly, mice overexpressing MEK7 also presented diastolic dysfunction and paradoxically high levels of ANF mRNA, which is considered a marker for cardiac hypertrophy [86]. The reduction in connexin 43 and gap junctions between ventricular cardiomyocytes might explain the absence of hypertrophy in the presence of increased ANF expression [86]. Also in the case of JNK, the exact mechanisms through which it regulates cardiac hypertrophy remains poorly determined.

### 4.3. p38 MAPK

In addition to JNK and ERK1/2, p38 MAPK was also intensively investigated. There are at least four isoforms of p38 MAPK that have been involved in cardiac remodeling and inflammation [87]. This signaling pathway is predominantly involved in the inflammatory response, and it can be activated by proinflammatory cytokines, chemokines, and hormones [88–90]. MEK 3, 4, and MEK 6 are the upstream kinases that directly activate p38MAPK [82]. Several downstream transcription factors have been identified as potential p38MAPK substrates, including activating transcription factor-1 (ATF-1), ATF-2, Elk-1, serum response factor (SRF), growth arrest, and myocyte enhance factor 2C (MEF 2C) [91–93]. Since a large amount of studies has been performed *in vitro* in neonatal rat cardiomyocytes, the role of p38 MAPK in cardiac clinical modifications remains to be confirmed. Nevertheless, p38 MAPK has been implicated in the regulation of both cardiac growth and hypertrophy [94]. Indeed, the inhibition of the p38 MAPK pathway via pharmacological inhibitors or adenovirus blunted the hypertrophic effect associated with p38 MAPK activation [94–96]. These hypertrophic effects of p38 MAPK phosphorylation in cardiomyocytes are also supported by another study where specific activation via adenovirus in ventricular muscle cells induced cardiac hypertrophy [84].

By contrast, Choukroun and coworkers have shown that p38 MAPK is not required for agonist-induced hypertrophy in cardiomyocytes [98]. To determine the role of the p38 MAPK pathway in the response to endothelin-1 (ET-1, a hypertrophic agent), neonatal rat cardiomyocytes were concomitantly treated with ET-1 and the selective p38 MAPK inhibitor, SB203580. The results revealed that this inhibitor had no effect on ET-1-induced hypertrophy, hence suggesting that p38 MAPK activation may not be required during cardiac cell hypertrophy [98]. Since these preliminary studies present some limitations (due to the use of the cardiomyocyte model [immature neonatal cells] and the modest specificity of pharmacological inhibitor, that can inhibit also other intracellular pathways), a genetic approach was also performed. Transgenic mice specifically overexpressing MEK 3 and MEK 6 (upstream activators of p38 MAPK) in the heart do not exhibit cardiac hypertrophy, despite the development of ventricular wall thinning and premature death with signs of congestive heart failure [99].

**Figure 1:**
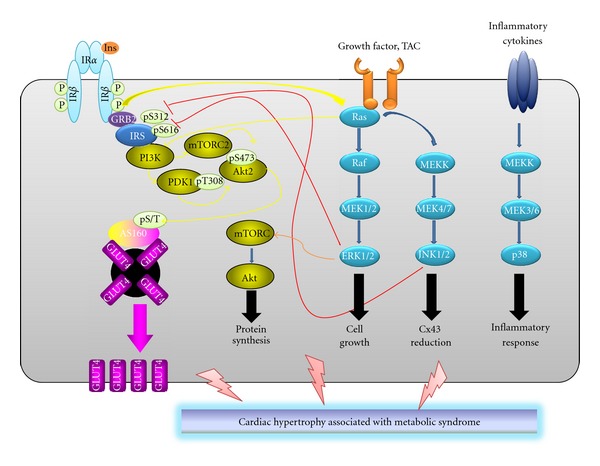
Intracellular pathways mediating hypertrophic cardiac remodelling in metabolic syndrome. Intracellular pathways mediated by phosphatidylinositol 3-kinase (PI3-K) activation play a pivotal role in insulin-mediated glucose transport in cardiomyocytes. Metabolic syndrome is associated with impaired intracellular signaling that could be activated by various stimuli, including inflammatory cytokines. These pathways, mainly dependent on MAPK activation (composed of extracellular signal-regulated kinase [ERK], c-Jun N-terminal kinase [JNK], and p38 MAPK), could be also triggered by chirurgical manipulation (such as transverse aortic constriction [TAC]). MAPK activation might be considered as a critical mechanism aggravating cardiac hypertrophy as well as cardiomyocyte insulin resistance in metabolic syndrome.

Taken together, these basic research studies suggest that p38 MAPK, ERKs, and JNKs may all be involved in promoting cardiac hypertrophy (Figure 1). In particular, p38 MAPK activation might also contribute to cardiac fibrosis, while ERK and JNK activation might promote cardiomyocyte growth and defects in gap junctions, respectively. The activation of a single MAPK-mediated cascade might not be sufficient to determine a clinically relevant adverse cardiac remodeling. 

## 5. Potential Role of MAPK in Metabolic Syndrome-Related Cardiac Adverse Remodeling

As suggested above, the alterations in cardiac MAPK activation might be induced in metabolic syndrome by insulin resistance and abnormal inflammation. The association between these molecular dynamics was so relevant that an altered MAPK activation pattern might also be a potential cause of hyperinsulinemia [100]. This pathophysiological role of MAPK in metabolic syndrome-induced heart remodeling was confirmed for JNK activation that was associated with both developments of insulin resistance and cardiac hypertrophy in metabolic syndrome [82, 101]. Assessing the role of ERKs in metabolic syndrome cardiac remodeling is much more complicated since ERK2-knockout mice are not viable [102, 103]. On the other hand, ERK1 knockout mice are viable and fertile. Thus, ERK1-deficient mice were investigated, and they were shown to be protected from diet-induced obesity and insulin resistance [104]. Nonetheless, mice lacking the ERK1 negative regulator p62 presented altered metabolism with increased adipogenesis, reduced energy expenditure, and reduced insulin sensitivity [105]. However, these animals were not investigated on concomitant cardiac remodeling and might be considered a good model to assess metabolic syndrome adverse heart remodeling. 

Inflammatory mediators such as TNF are elevated in metabolic syndrome [106]. Condorelli and coworkers showed that Akt and the JNK MAPK mediate TNF-induced hypertrophy in cultured cardiomyocytes [107]. TNF-mediated eccentric cardiac hypertrophy in response to intermittent hypoxia was shown to be mediated by ERK and STAT3 activation in adult rat myocardium [97]. On the other hand, IL-6 levels might also be involved in the determinism of cardiac hypertrophy [22]. Consistent with this hypothesis, Hirota and coworkers demonstrated a concomitant overexpression of both IL-6 and IL-6 receptor in a mouse model of cardiac hypertrophy [108]. Although these observations suggest a potential role for IL-6 in metabolic syndrome, the potential activation of intracellular signaling pathways by this cytokine remains unclear. Cardiotrophin-1 (CT-1), a newly discovered member of the IL-6 family and a key regulator of metabolism were also reported to be upregulated in metabolic syndrome [109, 110]. This cytokine was shown to favor cardiac hypertrophy and fibrosis in mice [23]. Thus, CT-1 might also underlie adverse remodeling in metabolic syndrome and thus represent an attractive pathophysiological target. 

Although evidence for the role of p38 MAPK in metabolic syndrome is still lacking (except for some animal studies in diabetic fibrotic cardiomyopathy) [111], enhanced MAPK signaling was shown to increase both insulin sensitivity and promote hypertrophic cardiac remodeling (Table 2). Thus, these proteins might represent a promising target to improve metabolic, inflammatory, and cardiac pathophysiology in metabolic syndrome.

## 6. Conclusions

In the past decades, relevant progresses have been made in the attempt to define the role of MAPKs in metabolic syndrome and in its clinical manifestations, including heart adverse pathophysiological remodelling. The heart structure has been described to be directly influenced by the mechanical stress, characterizing certain components of the metabolic syndrome (such as hypertension). Importantly, this adverse cardiac remodelling might be partially regulated by elevated hormones and inflammatory cytokines. MAPKs might represent the final pathways commonly activated within cardiomyocytes by both mechanical and soluble determinants. Despite some limitations due to animal and *in vitro* models, MAPK (mainly JNK and ERK) activation in the peripheral organs (including the heart) in metabolic syndrome was shown to induce insulin resistance and to increase inflammation. On the other hand, although the effect of p38 MAPK and JNK phosphorylation remains controversial, mounting evidence indicates ERK1/2 as responsible for promoting cardiomyocyte growth. We believe that MAPKs might be considered as potential therapeutic targets for drugs aimed to improve metabolic and cardiac dysfunctions in metabolic syndrome. Indeed several inhibitors of MAPK signaling proteins are currently available. Some of them are already being tested in clinical trials for oncological disorders and may theoretically find application also for the treatment or prevention of heart dysfunction in metabolic syndrome. Alternatively, strategies targeting downstream effectors/transcription factors in these cascades could also be a viable therapeutic option.

## Figures and Tables

**Table 1:** Different disorders in metabolic syndrome are associated with cardiac structural and functional changes.

Metabolic syndrome characteristic	Adverse cardiac remodelling	Reference	Method of assessment
	Obese women have higher end-diastolic septal and posterior wall thickness, left ventricle mass, and relative wall thickness than nonobese	[13]	Echocardiography and tissue Doppler imaging
Obesity	Uncomplicated severe obesity is associated with adapted and appropriate changes in cardiac structure and function	[14]	Echocardiography
	Reduced left ventricle systolic and diastolic function and increased myocardial reflectivity characterize obese patients as compared to referents	[15]	Transthoracic echocardiography, myocardial Doppler-derived systolic and early diastolic velocity, strain and strain rate imaging, and tissue characterization with cyclic variation and calibrated integrated backscatter

	Diabetes, fasting glucose, and fasting insulin levels are associated with left ventricular hypertrophy	[16]	Echocardiography and laboratory testing
Diabetes	Increased heart size in obese men	[17]	Autopsy
	Postmortem analysis of obese patients that died from gastric bypass complication revealed cardiac hypertrophy	[18]	Autopsy

Hypertension	Left ventricle mass is positively associated with the number of metabolic risk factors in normotensive and hypertensive participants	[19]	Echocardiography

	Increased left ventricular mass and reduce left ventricular relaxation	[20]	Echocardiography was used to assess pulse-wave Doppler and tissue Doppler imaging
Metabolic syndrome (defined as a cluster of all previously cited disorders [2])	Ventricular diastolic dysfunction, mean left ventricular mass, and left ventricular diameter significantly increase with the number of features of the metabolic syndrome	[21]	Structured clinical interview with a physician, ECG and a transthoracic M-mode, and 2D echocardiogram
	High levels of IL-6 that could be observed in metabolic syndrome induce cardiac fibrosis	[22]	Blood-perfused isolated heart
	Cardiotrophin-1 treatment, mimicking the upregulated level found in metabolic syndrome, induces cardiac fibrosis	[23]	Echocardiography, Doppler, and echo tracking device and *ex vivo* approach

**Table 2:** Role of MAPK activation in metabolic syndrome-associated cardiac hypertrophy.

Author and reference number	Year	Experimental model	MAPK phosphorylation	Cardiac remodeling
Okoshi et al. [62]	2004	Primary cultures of neonatal rat cardiomyocytes	Increased P-ERK1/2	Aldosterone induces hypertrophy through ERK1/2 activation

Yue et al. [63]	2000	Primary cultures of neonatal rat cardiomyocytes	Increased P-ERK1/2	ERK1/2 activation mediates endothelin-1- and phenylephrine-induced cardiac hypertrophy
Wang and Proud [64]	2002	Adult rat ventricular cardiomyocytes		

Chen et al. [97]	2007	Rat myocardium	Increased P-ERK5; P-STAT-3; P-p38 MAPK	Long-term intermittent hypoxia is associated to induced cardiac hypertrophy through the activation of MAPK pathways

Takeishi et al. [79]	2001	Guinea pigs	Increased P-ERK1/2, P-p38 MAPK	Chronic pressure-overload and acute mechanical stretch-induced cardiac hypertrophy is mediated by ERK1/2 and p38 MAPK activation

Esposito et al. [80]	2010	Mice left ventricle and white blood cells from mice and hypertensive patients with controlled blood pressure values	Increased P-ERK1/2, P-p38 MAPK, P-JNK	MAPKs are sensors of pressure overload

Rose et al. [82]	1998	Primary cultures of neonatal rat cardiomyocytes	Increased P-JNK and P-p38 MAPK	JNK activation induces hypertrophy, while concomitant activation of p38 MAPK and JNK inhibits hypertrophic response

Wang et al. [84]	1998	Primary cultures of neonatal rat cardiomyocytes	Increased P-JNK and P-p38 MAPK	JNK induces hypertrophy, while concomitant activation of p38 MAPK and JNK fails to promote hypertrophic response

Zechner et al. [96]	1997	Primary cultures of neonatal rat cardiomyocytes	Increased P-p38 MAPK	Transfection of the cell with constructs activating MAPKs revealed a central role of p38 MAPK activation in cardiac hypertrophy

Choukroun et al. [98]	1998	Primary cultures of neonatal rat cardiomyocytes	Increased SAPK and not P-ERK	In contrast to SAPK, ERK activation is not required for hypertrophic response induced by endothelin
